# Single-Retained Lithium Disilicate “Maryland” with Pontic-Derived Stamp Technique for Anterior Symmetry: A Case Report

**DOI:** 10.3390/dj14070427

**Published:** 2026-07-10

**Authors:** Pier Edoardo Maltagliati, Ahmed Deraz, Alberto Maltagliati, Giovanni Messina, Yashar Imenpour, Stefano Benedicenti

**Affiliations:** 1 Department of Endodontics and Restorative Dentistry, University of Genoa, 16132 Genoa, Italy; 2 Master’s Program in Advanced Esthetics and Restorative Dentistry, University of Genoa, 16132 Genoa, Italy; ahmedkamil910@gmail.com; 3 Department of Integrated Diagnostic and Surgical Sciences, University of Genoa, 16132 Genoa, Italy; 4 Independent Researcher, 25121 Brescia, Italy; 5 Department of Internal Medicine and Medical Specialties (DIMI), University of Genoa, 16132 Genoa, Italy; 6 Department of Surgical and Diagnostic Sciences, University of Genoa, 16132 Genoa, Italy

**Keywords:** maryland bridge, single-retainer cantilever, resin-bonded, lithium disilicate, CAD/CAM dentistry, stamp technique

## Abstract

**Background/Objectives:** Single-retainer Maryland adhesive restorations provide a minimally invasive fixed option for replacing missing anterior teeth in young patients. When congenital absence of a maxillary lateral incisor is associated with a contralateral conoid lateral incisor, the clinical problem extends beyond tooth replacement to bilateral symmetry management. This case report describes a digital-restorative workflow combining a single-retainer lithium disilicate Maryland adhesive restoration with a pontic-derived stamp technique for contralateral symmetry correction. **Methods**: A medically healthy 13-year-old patient presented with congenital absence of the maxillary left lateral incisor (FDI 22; Universal 10) after orthodontic treatment and had been wearing a removable appliance to replace the single missing tooth. The contralateral maxillary right lateral incisor (FDI 12; Universal 7) presented with conoid morphology, further compromising anterior symmetry. Clinical assessment confirmed ideal mesiodistal space at FDI 22, a vital and unrestored FDI 21 with intact enamel and healthy periodontal support, and the absence of abnormal overjet, overbite, or excursive contact pattern. Based on the findings, a single-retainer Maryland adhesive restoration was fabricated from lithium disilicate (IPS e.max CAD) and bonded to the maxillary left central incisor (FDI 21; Universal 9) using an enamel-only preparation and adhesive cementation protocol. The fixed restoration was combined with a pontic-derived stamp workflow to guide direct composite reshaping of the contralateral conoid lateral incisor. **Results:** At 1-year follow-up, no debonding, sensitivity, marginal discoloration, or soft-tissue inflammation was observed. The contralateral composite reshaping remained clinically stable, and the patient and guardian reported improved comfort and esthetic satisfaction compared with the previous removable appliance. **Conclusions**: This case suggests the short-term clinical feasibility of combining a single-retainer lithium disilicate Maryland adhesive restoration with a pontic-derived stamp workflow to achieve minimally invasive tooth replacement and contralateral symmetry correction. Longer follow-up and broader case series are required to confirm the long-term predictability and reproducibility of this approach.

## 1. Introduction

Replacing a missing maxillary lateral incisor in the esthetic zone often requires balancing appearance with maximal tooth preservation. In adolescents, implant placement can be postponed because continued dentofacial growth can lead to infraposition and esthetic complications over time [1]. When a patient also wants to avoid a bulky removable appliance for a single missing tooth, a conservative fixed adhesive solution becomes particularly attractive [2].

Single-retainer Maryland adhesive restorations provide a minimally invasive enamel-preserving alternative with favorable evidence for cantilever designs in the anterior region [2,3,4,5,6,7]. Digital workflows also allow standardization of contour planning, emergence profile, and adhesive restoration design while maintaining conservative preparation principles [8]. In young patients, this approach is particularly relevant because it allows restoration of the missing tooth while preserving future treatment options and minimizing intervention on healthy dental tissues [9,10].

In the present case, the challenge was not limited to replacement of the missing left lateral maxillary incisor. The contralateral lateral incisor presented with a conoid morphology, creating a second esthetic problem that affected the overall balance of the smile. Consequently, the clinical objective extended beyond the replacement of the missing tooth and required simultaneous management of bilateral anterior symmetry. This report therefore describes an integrated restorative strategy in which a digitally planned lithium disilicate single-retainer Maryland adhesive restoration was combined with a pontic-derived stamp technique to guide contralateral composite reshaping and improve anterior symmetry. The distinguishing feature of the present report is that the definitive pontic morphology itself was used as the reference form for the direct reshaping of the opposite lateral incisor, thereby linking prosthetic replacement and esthetic correction within the same restorative concept. In this context, the stamp technique refers to the use of a transparent matrix generated from a digitally planned tooth morphology to guide direct composite placement and reproduce the intended contour with reduced freehand variability [11,12].

In addition to tissue preservation, single-retainer adhesive restorations are particularly relevant in young patients because they provide a fixed solution while maintaining future restorative flexibility. Their conservative nature makes them suitable when preservation of intact enamel is a priority and when long-term treatment planning must remain adaptable. Recent evidence has also supported the use of cantilever resin-bonded fixed restorations in the anterior region, especially when case selection and adhesive execution are carefully controlled [2,8]. In the present case, the restorative challenge was further increased by the presence of a contralateral conoid lateral incisor, meaning that successful treatment required not only replacement of the missing tooth but also harmonization of bilateral anterior form and symmetry [11,12].

## 2. Case Report

**Figure 1:**
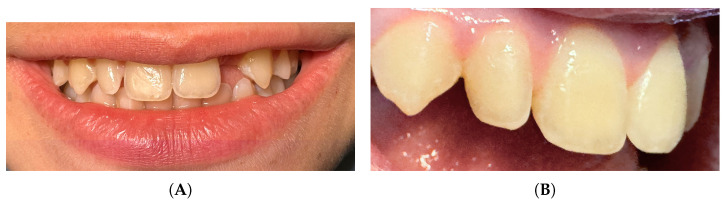
(**A**) Pre-operative frontal view showing missing FDI 22 and anterior asymmetry. (**B**) Pre-operative view highlighting the adjacent tooth morphology.

**Figure 2:**
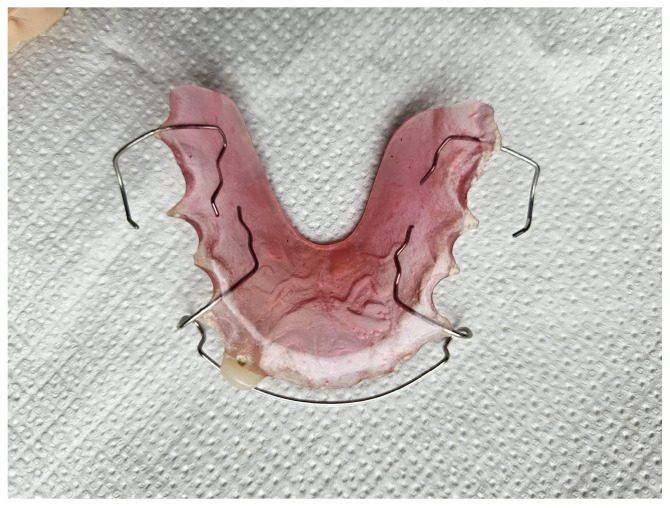
Previous removable appliance used.

A medically healthy 13-year-old patient presented after completion of orthodontic treatment with a congenitally missing maxillary left lateral incisor (FDI 22; Universal 10). The patient had previously received a removable appliance to replace the missing tooth, but this was considered uncomfortable and undesirable for daily use. Clinical examination confirmed the edentulous space at FDI 22 and a contralateral conoid/peg-shaped maxillary right lateral incisor (FDI 12; Universal 7), resulting in marked anterior asymmetry (Figure 1A,B and Figure 2).

Diagnostic evaluation showed that the mesiodistal space at FDI 22 was ideal after orthodontic treatment. FDI 21 was vital, unrestored, and clinically suitable as an adhesive abutment, with intact enamel and healthy periodontal support. No abnormal overjet, overbite, or excursive contact pattern was identified. The cantilever restoration was therefore planned to remain out of occlusion in maximum intercuspation and during mandibular excursions.

The treatment objective was divided into two parts: first, to provide a fixed conservative replacement for the missing lateral incisor while preserving sound enamel on the abutment tooth; second, to correct the contralateral conoid morphology and improve bilateral anterior symmetry. Given the patient’s age, recent completion of orthodontic treatment, the desire to discontinue use of the removable appliance, the favorable enamel condition of the abutment tooth, and the need to simultaneously address the contralateral lateral incisor, a single-retainer Maryland adhesive restoration combined with pontic-guided composite reshaping was selected. Growth-related treatment planning also considered the patient’s developmental status. As the patient was 13 years old and residual craniofacial growth could not be reliably excluded, implant-supported rehabilitation was deferred until growth completion. Consequently, a minimally invasive adhesive fixed solution was chosen to provide functional and esthetic rehabilitation while preserving future restorative options.

From a clinical standpoint, this treatment plan was favored over more invasive alternatives because it allowed a fixed solution in a young patient while preserving enamel on the abutment tooth and avoiding full-coverage preparation. At the same time, it offered a practical method to harmonize the morphology of the opposite lateral incisor, which would otherwise have remained visibly inconsistent with the prosthetic replacement.

## 3. Clinical Procedures

### 3.1. Digital Planning and Prosthesis Design

**Figure 3:**
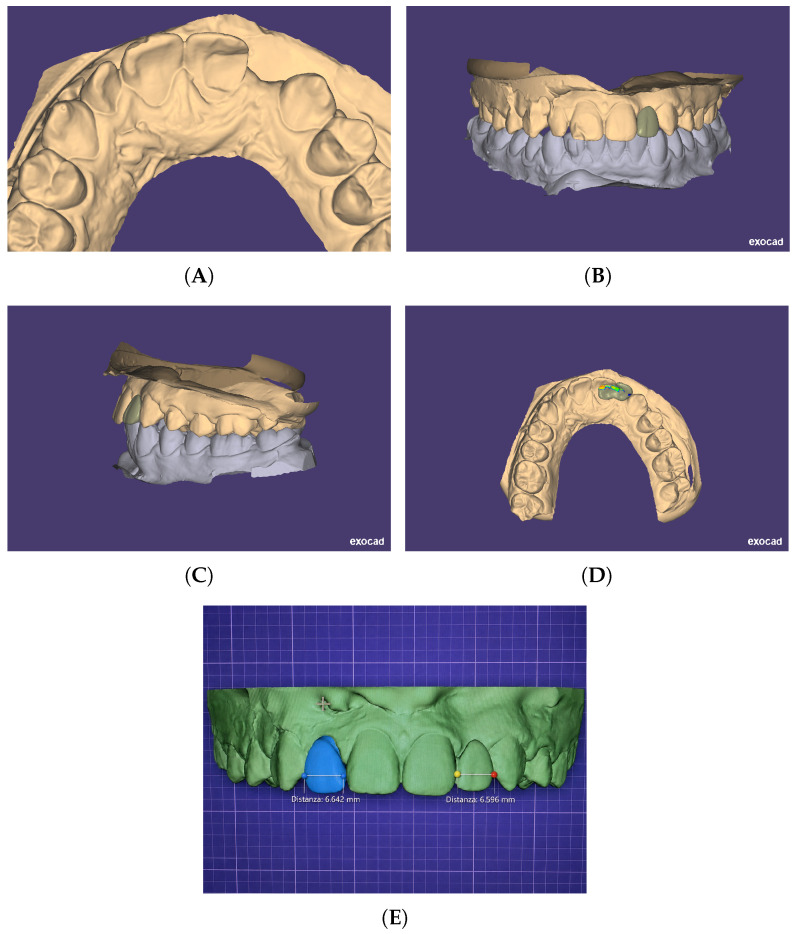
(**A**) Digital design showing the bonding surface and wing extension. (**B**) Frontal design view. (**C**) Side view. (**D**) Occlusal contact-map view. (**E**) Digital CAD width measurements for bilateral symmetry planning.

A lab-assisted CAD/CAM workflow was used. An intraoral scan was acquired using an Aoralscan 3 intraoral scanner (SHINING 3D Dental), and a single-retainer Maryland adhesive restoration design was created in Exocad software by a laboratory technician to replace FDI 22 with palatal bonding to FDI 21. Digital contact and contour evaluation were performed prior to fabrication (Figure 3A–D). The restoration was produced from lithium disilicate ceramic (IPS e.max CAD) and delivered for clinical bonding.

Digital CAD measurements were recorded from the frontal CAD design to support symmetry planning (Figure 3E). The pontic of the single-retainer Maryland adhesive restoration replacing FDI 22 had a mesiodistal width of 6.642 mm, while the contralateral lateral-incisor reference width used for stamp-guided reshaping was 6.596 mm. These values were used as basic digital reference parameters for harmonizing the bilateral lateral-incisor proportions.

The digital planning stage was central to the case because it served both prosthetic and esthetic purposes. It allowed definition of the retainer extension, evaluation of contour and occlusal relationships, and generation of the morphology later transferred to the contralateral lateral incisor through the stamp-guided composite workflow. In this respect, the digital design phase did not merely precede fabrication of the restoration, but also informed the final esthetic integration of the anterior segment.

### 3.2. Abutment Preparation (FDI 21; Universal 9)

The preparation of FDI 21 was limited to enamel and consisted of approximately 0.5 mm palatal reduction, controlled using a 0.5 mm depth-cutting bur and checked intraorally with a WHO periodontal probe. The reduction was also verified during the digital laboratory design to support an enamel-only preparation. Enamel roughening was performed using a fine red-coded football-shaped diamond bur to enhance micromechanical retention (Figure 3A). No dentin exposure was intended or clinically observed.

This enamel-restricted design was selected to preserve tooth structure and optimize adhesive predictability. In the context of a young patient, maintenance of an enamel-based bonding substrate was considered particularly advantageous because it supported a conservative treatment philosophy while avoiding unnecessary biological sacrifice.

### 3.3. Isolation

**Figure 4:**
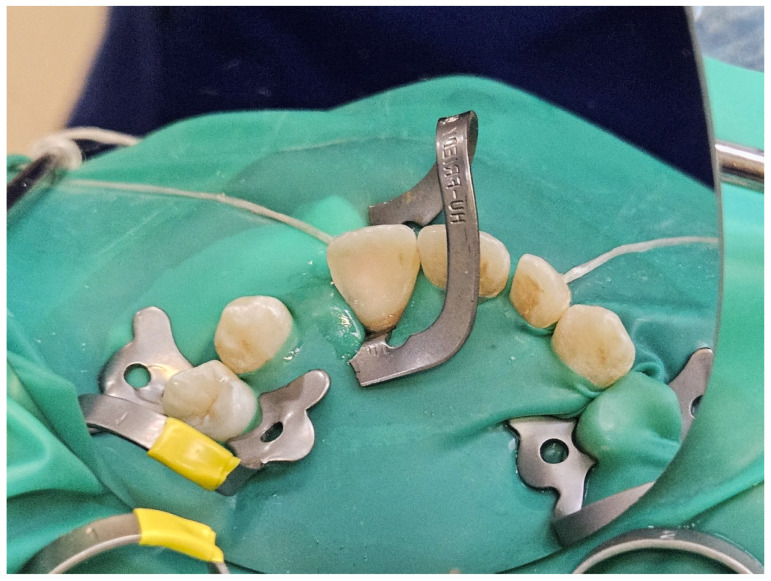
Rubber dam isolation with sectioned 212 clamp, floss, PTFE, and posterior clamps (1, 2A).

Rubber dam isolation was achieved using floss ligatures and clamp stabilization to maintain a dry bonding field. A 212 retraction clamp was sectioned to create a single-wing configuration, improving anterior access while maintaining soft-tissue retraction. Floss ligatures were placed around the 21/22 region to support soft-tissue management and facilitate cement clean-up. Additional posterior clamp support was provided using premolar clamps 1 and 2A. PTFE tape (Teflon) was used to further seal the mesial and distal aspects and reinforce isolation (Figure 4).

Strict isolation was considered essential because both the adhesive bonding of the lithium disilicate restoration and the subsequent direct composite reshaping procedure depended on reliable moisture control. The modified isolation setup was therefore used to improve access, visibility, and control during the critical adhesive stages of treatment.

### 3.4. Adhesive Cementation of the Single-Retainer Lithium Disilicate Maryland Adhesive Restoration

Before isolation, a try-in was performed to verify complete seating and marginal adaptation, and the fit was clinically acceptable. Rubber dam isolation was then established.

The inner surface of the lithium disilicate restoration was cleaned using a pumice slurry (pumice + water) with a surfactant to remove contaminants and activate the ceramic surface, then rinsed and air-dried. The bonding surface was etched with 5% hydrofluoric acid for 30 s, thoroughly rinsed, and dried. Then 37% orthophosphoric acid was applied for 30 s, followed by rinsing and air-drying. A silane coupling agent (Monobond Plus) was applied for 60 s and air-dried.

The enamel surface of the abutment was selectively etched with 37% phosphoric acid for 30 s, rinsed and gently air-dried. A universal adhesive (Ivoclar Universal Adhesive) was applied according to the manufacturer’s instructions. The restoration was bonded using dual-cure resin cement (Variolink Esthetic DC), and excess cement was carefully removed using microbrushes/explorers and dental floss interproximally. The final excess removal at the margins was refined using a No. 12 scalpel blade (Bard-Parker 12) before final curing. Light curing included tack curing for 5 s to facilitate excess removal, followed by 30 s on each of the four sides.

After bonding, the occlusion was rechecked and adjusted so that the cantilever remained free of centric and excursive contacts. Occlusal verification was performed using articulating paper in maximum intercuspation and during protrusive and lateral excursions; the restoration was adjusted until no contact marks were detected on the cantilevered pontic or retainer.

Maintenance of the restoration out of occlusion was considered a key clinical objective of the prosthetic phase, as it reduced mechanical stress on the retainer and supported the adhesive rationale of the treatment.

### 3.5. Pontic-Derived Stamp Technique for Contralateral Reshaping (FDI 12)

**Figure 5:**
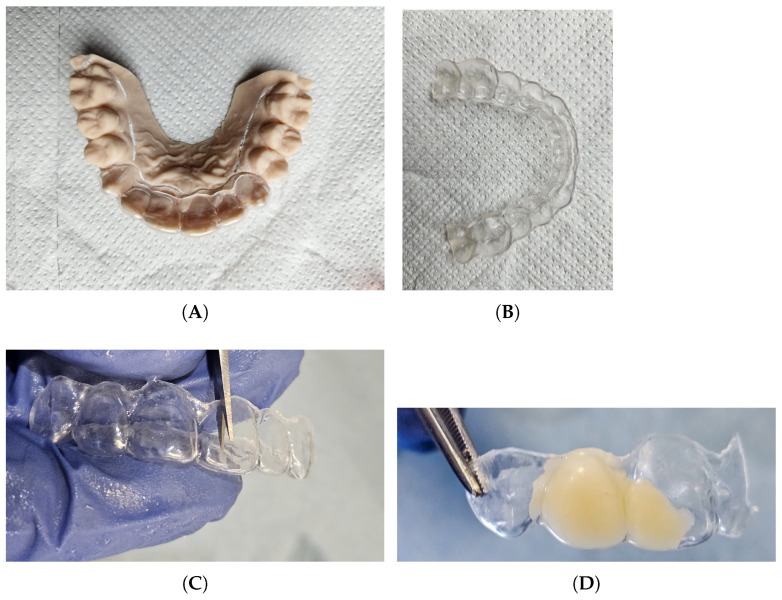
(**A**) Printed model mock-up with pontic-derived contour reference for FDI 12 reshaping. (**B**) Thermoformed clear stamp matrix adapted from the printed model. (**C**) Selective trimming of the stamp matrix to reproduce the planned contour. (**D**) Composite resin placed within the matrix to guide direct reshaping.

Anterior symmetry was refined using a stamp-guided composite technique derived from the planned pontic morphology. A clear retainer-like matrix was fabricated using ERKODUR disks (1.00 mm) (ERKODENT) and trimmed and adapted to reproduce the intended contour (Figure 5A–D). The stamp matrix was generated from the software-designed mock-up/wax-up, and the retainer-like matrix was thermoformed on the printed model derived from the CAD/CAM pontic form. The matrix was checked intraorally and positioned on FDI 12 to guide contouring.

**Figure 6:**
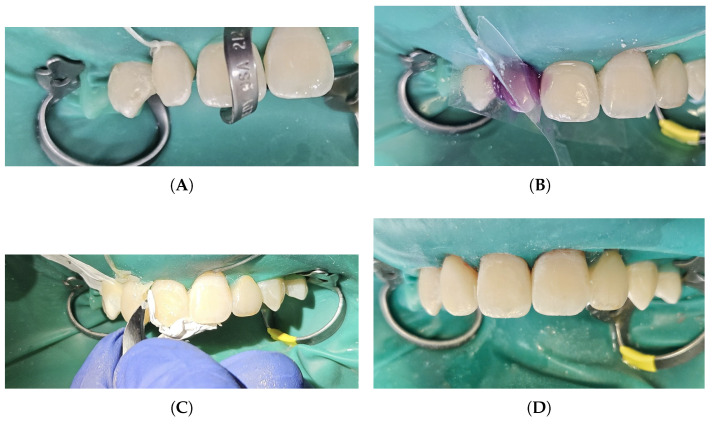
(**A**) Isolation under rubber dam. (**B**) Enamel etching. (**C**) Contour refinement with No. 12 blade. (**D**) Immediate result after reshaping.

After isolation, enamel was etched for 30 s, rinsed, and dried (Figure 6A,B). The same universal adhesive (Ivoclar Universal Adhesive) was applied according to the manufacturer’s instructions. Composite resin (Estelite) was placed and shaped using matrix guidance and light-cured while maintaining adaptation. Excess material was removed and contour refined using a No. 12 scalpel blade (Bard-Parker 12) (Figure 6C), and the immediate result under rubber dam was documented (Figure 6D). Finishing and polishing were completed using a fine-grit, red-band diamond polishing instrument (KENDA Nobilis, COLTENE/KENDA), followed by a flame-shaped silicone rubber polishing point (KENDA Maximus, COLTENE/KENDA). Interproximal finishing was completed using Profin finishing strips (Komet Dental) to refine proximal anatomy and surface continuity.

This stamp-guided workflow reduced freehand variability and allowed transfer of the definitive prosthetic morphology to the opposite lateral incisor in a controlled manner. As a result, the final symmetry correction was guided by the planned pontic anatomy itself rather than by visual approximation alone.

### 3.6. Outcome and Follow-Up

At 1-year follow-up, the single-retainer Maryland adhesive restoration remained intact with no debonding, sensitivity, marginal discoloration, or soft-tissue inflammation, and the contralateral composite reshaping remained clinically stable. The patient and guardian reported improved comfort and esthetic satisfaction compared with the previous removable appliance. The findings indicated satisfactory short-term behavior of both the adhesive prosthetic component and the direct composite reshaping.

### 3.7. Timeline

**Figure 7:**
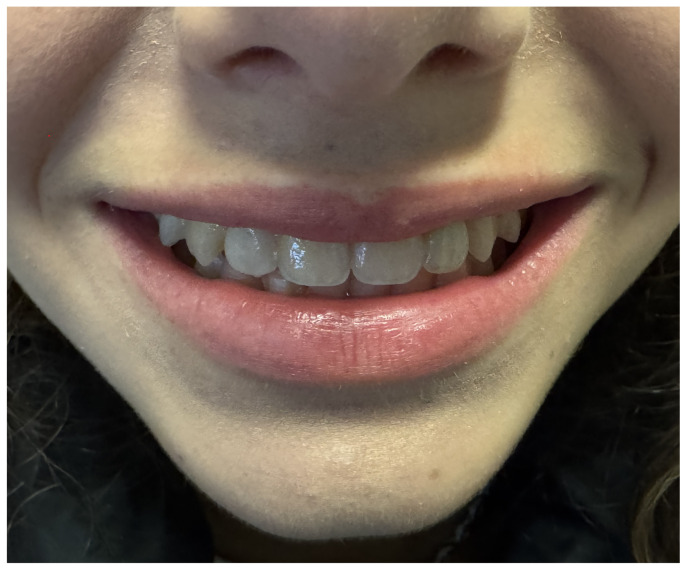
Final smile showing improved anterior symmetry.

Orthodontic treatment completion → restorative diagnosis and digital planning → adhesive cementation of the single-retainer Maryland adhesive restoration and contralateral stamp-guided composite reshaping → 1-year follow-up (Figure 7).

## 4. Discussion

This case illustrates a conservative approach for management of a congenitally missing maxillary lateral incisor using a single-retainer Maryland adhesive restoration while simultaneously correcting a contralateral conoid lateral incisor. The importance of the case lies in this dual indication: the objective of the treatment was not only replacement of the missing tooth, but also restoration of bilateral anterior symmetry through controlled reshaping of the opposite lateral incisor.

The single-retainer cantilever design was selected to maintain an enamel-limited preparation and avoid full-coverage restorations. Several alternative options could be considered, including a ceramic veneer or full-coverage restoration for FDI 12, continued removable prosthetic replacement, or future implant-supported rehabilitation for FDI 22. However, in this young patient, veneer or crown preparation was not preferred because it would require greater irreversible removal of sound tooth structure, while implant therapy was deferred due to growth-related considerations. The selected adhesive approach therefore provided a conservative, economically accessible, fixed solution while preserving future restorative options. In the present case, the absence of abnormal anterior contacts and the ability to keep the single-retainer Maryland adhesive restoration out of occlusion further supported this treatment choice [3,4,5,6,7,9,13].

Lithium disilicate was used because it offered favorable esthetic integration in the anterior region together with an established adhesive protocol. Although zirconia-based cantilever resin-bonded fixed dental prostheses have stronger long-term clinical evidence, lithium disilicate was selected in this case because of its favorable optical integration in the anterior region, etchable glass-ceramic surface, and compatibility with hydrofluoric acid/silane-mediated adhesive bonding. In addition, lithium disilicate has been investigated in thin veneer configurations and 0.5 mm preparation depths, supporting its use in minimally invasive anterior restorative situations when adhesive bonding and occlusal control are carefully managed. Recent clinical evidence has also reported favorable short-term outcomes for lithium disilicate cantilever resin-bonded fixed dental prostheses replacing missing maxillary incisors. Therefore, lithium disilicate was considered suitable for this carefully selected short-span anterior restoration, provided that strict adhesive protocol and occlusal control were maintained [13,14,15].

For symmetry management, the pontic-derived stamp matrix reduced freehand variability by transferring the planned tooth form to the contralateral lateral incisor. Template-guided reshaping techniques have been described as reliable methods to transfer planned morphology to direct restorations in a controlled manner [11,12]. The key distinctive feature of this report is that the stamp morphology was derived directly from the digitally planned pontic form, allowing the definitive restorative design itself to guide the contralateral reshaping. This created a strong visual and morphological relationship between the replacement of the missing tooth and the correction of the lateral conoid.

From a broader clinical perspective, the case also illustrates the value of integrating digital planning with conservative adhesive dentistry in a way that extends beyond prosthetic replacement alone. Rather than treating the missing lateral incisor and the conoid contralateral lateral as separate esthetic problems, the final restorative design was used as a unifying reference for bilateral symmetry. This may be particularly useful in selected young patients in whom treatment must remain minimally invasive while still providing a coherent anterior result [9,10].

The principal limitations of this report are its single-case design, the short 1-year follow-up, and the absence of quantitative esthetic analysis. Therefore, longer follow-up is needed to further evaluate the clinical performance of this treatment approach. In addition, esthetic improvement was documented clinically and photographically, and patient-reported satisfaction was recorded; however, no standardized digital smile analysis was performed. Minor differences in photographic standardization may also be present, and detailed CAD/CAM design parameters were not prospectively recorded. Future studies with larger cohorts and longer follow-up are needed to further assess these findings.

## 5. Conclusions

Within the limitations of a single-case report and a 1-year follow-up, this case supports the short-term clinical feasibility of combining a single-retainer lithium disilicate Maryland adhesive restoration with a pontic-derived stamp workflow for conservative replacement of a congenitally missing maxillary lateral incisor and simultaneous contralateral symmetry correction. The workflow provided a fixed, minimally invasive solution while preserving enamel and maintaining future restorative options. These findings should be interpreted as preliminary and short-term; however, they suggest that this combined digital and adhesive workflow may be a useful conservative option in carefully selected young patients. Further clinical documentation with standardized outcome assessment, radiographic follow-up, evaluation of periodontal and occlusal stability, patient-reported outcomes, and longer observation periods is warranted.

## Data Availability

The data presented in this case report are available from the corresponding author upon reasonable request. Public sharing is restricted due to patient privacy and ethical considerations.
